# Common dependence on stress for the statistics of granular avalanches and earthquakes

**DOI:** 10.1038/srep12280

**Published:** 2015-07-21

**Authors:** Takahiro Hatano, Clément Narteau, Peter Shebalin

**Affiliations:** 1 Earthquake Research Institute, University of Tokyo, 113-0032 Tokyo, Japan; 2 Institut de Physique du Globe de Paris, Sorbonne Paris Cité, Univ Paris Diderot, UMR 7154 CNRS, 1 rue Jussieu, 75238 Paris, Cedex 05, France; 3 Institute of Earthquake Prediction Theory and Mathematical Geophysics, Profsoyuznaya str., 84/32, Moscow 117997, Russia

## Abstract

Both earthquake size-distributions and aftershock decay rates obey power laws. Recent studies have demonstrated the sensibility of their parameters to faulting properties such as focal mechanism, rupture speed or fault complexity. The faulting style dependence may be related to the magnitude of the differential stress, but no model so far has been able to reproduce this behaviour. Here we investigate the statistical properties of avalanches in a dissipative, bimodal particulate system under slow shear. We find that the event size-distribution obeys a power law only in the proximity of a critical volume fraction, whereas power-law aftershock decay rates are observed at all volume fractions accessible in the model. Then, we show that both the exponent of the event size-distribution and the time delay before the onset of the power-law aftershock decay rate are decreasing functions of the shear stress. These results are consistent with recent seismological observations of earthquake size-distribution and aftershock statistics.

Individual earthquakes may be regarded as large scale ruptures involving a wide range of structural and compositional heterogeneities in the crust. However, the statistical properties of a population of earthquakes are often described by simple power-laws. Among them, two laws are ubiquitous and occupy a central position in statistical seismology: the Gutenberg-Richter (GR) law^1^ and the Modified Omori law (MOL)^2^,^3^. The GR law describes the earthquake magnitude-frequency distribution


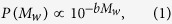


where *M*_*w*_ is the moment magnitude and *b* a constant with a value around 1 along active fault zones. The MOL describes the aftershock occurrence rate


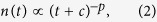


where *t* is the elapsed time from the triggering event (the so-called mainshock), *p* a positive non-dimensional constant with a typical value of 1 and *c* a time constant. The parameters in these two laws are believed to bear some information on the physical state of the crust. Indeed, *Schorlemmer et al*. find that the *b*-value of the GR law is decreasing going from normal (extension) over strike-slip (shear) to thrust (compression) earthquakes^4^. *Narteau et al*. also find that the time constant *c* in the MOL has the same dependence on the faulting mechanism^5^. These two observations indicate that, under a simple assumption, *b* and *c* are decreasing functions of shear stress. Although the underlying mechanism needs further investigation, this may reflect a common time-dependent behaviour of fracturing in rocks during the propagation of earthquake ruptures and the nucleation of aftershocks.

Because the shear stress along an active fault is not directly measurable, a solution to address stress dependences in earthquake statistics is to analyse models that implement a restricted set of physical processes. Such models are indeed numerous, ranging from rock fracture experiments^6^,^7^,^8^,^9^ to computer simulations on cellular automata^10^,^11^. Among them, sheared granular media^12^,^13^,^14^,^15^,^16^,^17^,^18^,^19^ are simple representations of granular fault gouges (Fig. 1), which are commonly used in geophysics to analyse deformation of highly damaged rocks in fault zones^20^,^21^,^22^. Additionally, both the energy and the stress can be easily defined in these models.

Here we use the simplest form of structural complexity - a bimodal distribution of particle size - to elucidate its fundamental control on the emergent scaling laws. We then perform numerical simulations showing that, as for real seismicity, avalanches in sheared granular matter obeys the GR law and the MOL with *b* and *c*-values being decreasing functions of the shear stress. The details of our numerical model are described in the Methods section.

## Results

### Characterisation of avalanches

**Figure 2:**
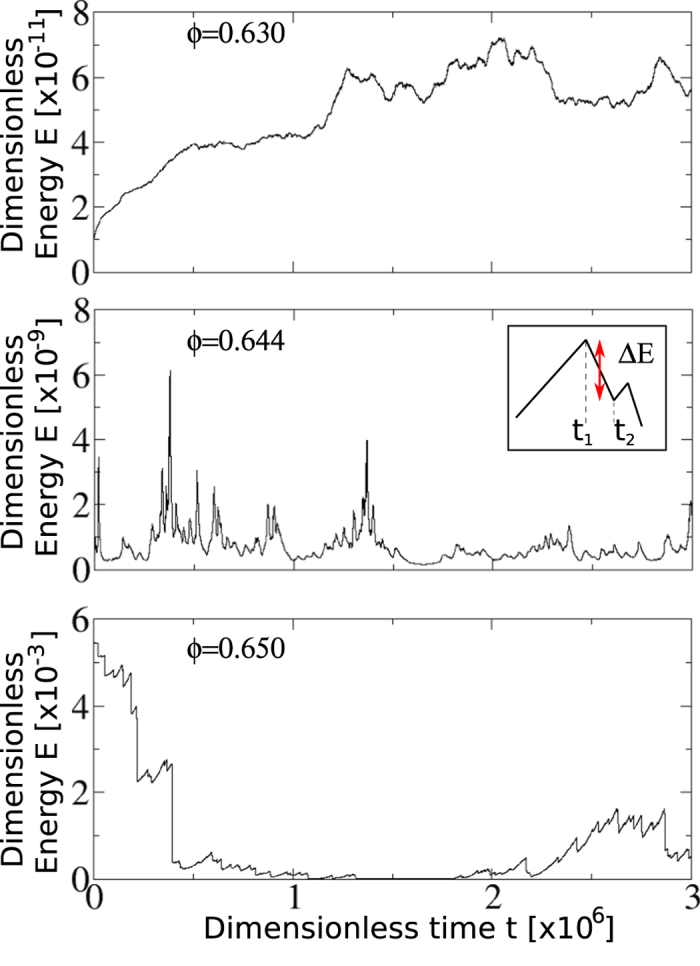
Typical time series of the elastic energy for a constant shear rate 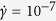 and different volume fraction *ϕ*: (top) *ϕ* = 0.630, (middle) *ϕ* = 0.644, (bottom) *ϕ* = 0.650. As shown by the normalization constant of the vertical axes, the total energy and the energy releases explore different ranges of magnitude according to volume fraction. Inset in the middle panel shows how we estimate the energy release associated with a single avalanche.

As observed in many previous works on amorphous particulate systems^14^,^15^,^16^,^17^,^20^,^21^,^23^,^24^,^25^,^26^,^27^,^28^,^29^,^30^, Fig. 2 shows that the temporal fluctuations of the energy becomes volatile if the shear rate is sufficiently low and the volume fraction is sufficiently high. Under such condition the kinetic energy is negligible in comparison with the elastic energy *E*(*t*), and therefore the elastic energy drop should approximate a transition from a local maximum to a local minimum in configurational energy. Then, an avalanche is defined as an abrupt drop of the elastic energy, *E*(*t*_1_) − *E*(*t*_2_), where *t*_1_ and *t*_2_ denote the beginning and the end of an event, respectively. This is illustrated in the inset of Fig. 2b.

Following the literature of earthquake studies^31^, we define the magnitude of avalanche as *M* ∫ log_10_[*E*(*t*_1_) − *E*(*t*_2_)] + *m*_0_, where *m*_0_ = 11 is chosen so that the magnitude of the smallest avalanches is approximately zero. Note that, with this definition of magnitude, the GR law reads *P*(*M*) ∝ 10^−2/3*bM*^, as the moment magnitude for earthquake^31^ is defined as *M*_*w*_ ≃ 2/3 log_10_[*E*(*t*_1_) − *E*(*t*_2_)] + const. Hereafter, we use *β* ∫ 2/3*b* instead of *b*. Other important quantities that characterise an avalanche are the initial stress 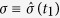 and the stress drop 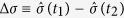. The definition of the shear stress and other technical remarks on avalanches are described in the Methods section.

### Magnitude-frequency distribution

**Figure 3:**
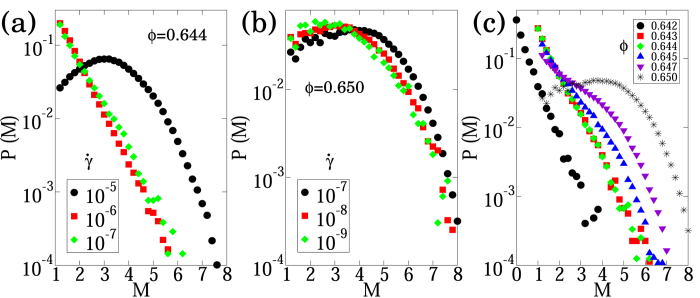
Avalanche magnitude-frequency distributions: (**a**) for a volume fraction *ϕ* = 0.644 and three shear rate values, 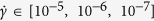. (**b**) for a volume fraction *ϕ* = 0.650 and three shear rate values, 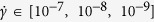. (**c**) for several volume fraction values at low shear rate, 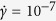 for *ϕ* ≤ 0.645 and 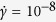 for *ϕ* > 0.645.

First, we discuss the nature of the avalanche magnitude-frequency distribution with respect to the two control parameters, the shear rate 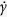 and the volume fraction *ϕ*. Figure 3a,b show these distributions at several shear rates for *ϕ* = 0.644 and *ϕ* = 0.650, respectively. Both parameters control the shape of the magnitude-frequency distribution. For example, one can observe a break in scale-invariance for high shear rates at low volume fraction (*ϕ* = 0.644 and 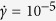 in Fig. 3a). Similarly, characteristic-size distribution (i.e, peaked at a single magnitude) are observed for high volume fraction (Fig. 3b). However, the distribution is independent of the shear rate below a characteristic 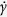-value, which may be interpreted as the inverse of the structural relaxation time. Not surprisingly, this threshold value is a decreasing function of the volume fraction. In the volume fraction range investigated here, it is approximately 10^−6^ for *ϕ* = 0.644 (Fig. 3a) and 10^−8^ for *ϕ* = 0.650 (Fig. 3b). Hereafter, we discuss such rate-independent behaviours by choosing sufficiently low shear rates.

Figure 3c shows rate-independent magnitude-frequency distributions at several volume fractions. They may be regarded as the GR law in the proximity of a critical volume fraction (*ϕ* ≃ 0.644), whereas they no longer obey the GR law at higher volume fractions. At much lower volume fractions (*ϕ* < 0.64), we can hardly obtain a sufficient number of avalanches that ensures statistical significance. It should be noted that the *β*-value in the proximity of a critical volume fraction is sensitive to the minute changes in volume fraction. The *β*-value is a decreasing function of the volume fraction ranging from 0.47 to 0.78. We obtain the smallest value (0.47) at the largest volume fraction (*ϕ* = 0.645) and the largest value (0.78) at the smallest volume fraction (*ϕ* = 0.642). The *β*-value at intermediate volume fractions (*ϕ* = {0.643, 0.644}) is approximately 0.64. This range of values is comparable to that of earthquakes^4^ for which the *β*-value varies from 0.50 along normal faults (i.e., extensional regime, low stress) to 0.73 along reverse faults (i.e., compressional regime, high stress).

We remark that distribution functions of other quantities also obey power laws. Among them, we find that the distribution functions of the avalanche duration, *t*_2_ − *t*_1_, exhibit power-law tails, the exponent of which is approximately 3.0 irrespective of the volume fraction. We also find that the exponent for the stress drop distribution is twice larger than that for energy drop.

As similar power law behaviours have been observed in many particulate systems, it is interesting to compare the present result with other studies on sheared granular matter. Actually, a range of *β*-value have been obtained: 0.36 to 0.95^13^, 0.82 to 0.89^14^, and 1.0^15^ have been reported. On the other hand, the GR law is not observed in an experiment that is conducted at lower volume fractions^16^. All these behaviours are consistent with the present result, where the *β*-value depends on both the volume fraction and the stress level. Thus, the *β*-value should not be an analogue for critical exponents. Some studies also report similar *β*-value variations^17^,^18^,^19^.

As an evidence of such non-universality, one can further illustrate a wide range of *β*-values obtained in various amorphous systems^23^,^24^,^25^,^26^,^27^,^28^,^29^,^30^. In a fracture experiment^32^ and a frictional system^33^, the *β*-values are also found to be sensitive to control parameters. There may exist many unknown ingredients that control the *β*-value, and further investigation is required for the unified understanding of all these exponents in general amorphous systems.

**Figure 4:**
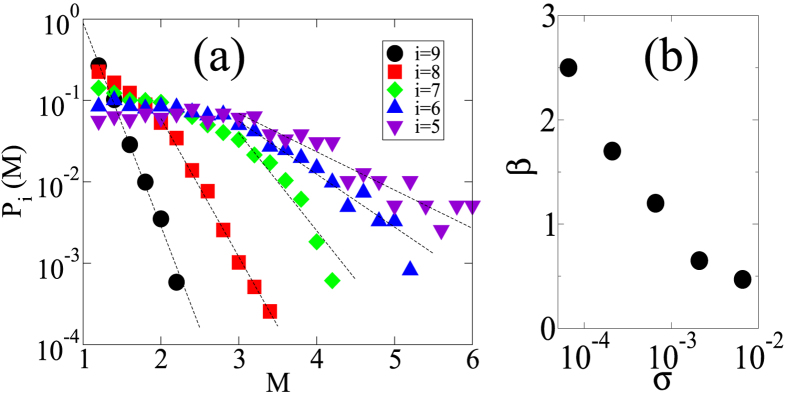
Dependency of the avalanche magnitude-frequency distribution on the global shear stress. (**a**) The avalanche magnitude-frequency distributions for different ranges of shear stress value, a volume fraction *ϕ* = 0.643 and a shear rate 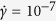. Events *i* are classify according to the value of the global shear stress at the inititation of the avalanches (See text for the exact ranges of shear stress value). (**b**) The slope of the magnitude-frequency distribution with respect to the global shear stress. The *β*-value is a decreasing function of the global shear stress.

To discuss the effect of shear stress on the *β*-value, we introduce the shear stress *σ* at the beginning of an event as an additional argument to the magnitude-frequency distribution. Then, *P*(*M*, *σ*) is the conditional probability of observing a magnitude *M* avalanche under the (global) shear stress value *σ*. For convenience, *σ* is integrated in a certain interval *S*_*i*_ ∫ [10^−*i*/2^, 10^−*i*/2+0.5^] (*i* = {1, 2, ···, 9}). Thus, we obtain the following distribution function: 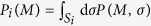. Figure 4a shows the behaviours of *P*_*i*_(*M*) at *ϕ* = 0.643. We can see that the probability of observing a larger avalanche increases as the shear stress increases. More importantly, the distribution function at each stress level develops a power-law tail with a *β*-value, which is a decreasing function of the shear stress (Fig. 4b). We confirm that this stress dependence is independent of the volume fraction in the range 0.642 ≤ *ϕ* ≤ 0.645. In addition, the shear stress dependence of the *β*-value is qualitatively the same as that in rock fracture experiments^7^.

### Aftershock statistics and the Modified Omori Law

**Figure 5:**
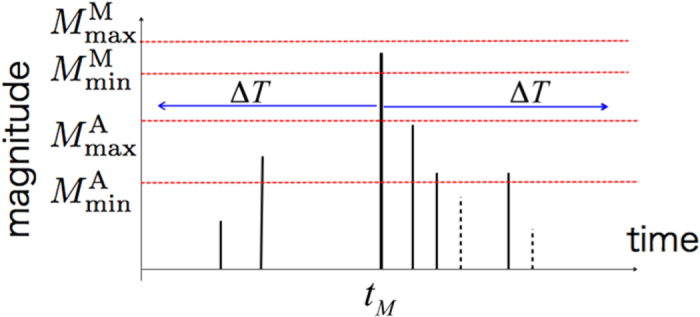
The declustering method to select mainshock and aftershocks. We consider two non-overlapping magnitude ranges for mainshocks 
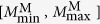
 and aftershocks 
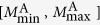
. A magnitude 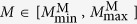 event occuring at time *t*_M_ is selected as a mainshock if there is no larger event in the time interval [*t*_M_ − Δ*T*; *t*_M_ + Δ*T*]. All magnitude 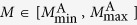 events in the time window [*t*_M_, *t*_M_ + Δ*T*] are selected as aftershocks (see also the Methods section.)

Second, we can also study time series of avalanches from our simulations to take them as analogues for mainshock-aftershocks sequences. Unlike the magnitude-frequency distribution, and despite the systematic occurrence of aftershocks in seismogenic areas, there are only few examples of such mainshock-aftershocks sequences in amorphous systems^9^,^14^. Triggered events may be difficult to distinguish from other events or simply too rare in individual sequences to exhibit a specific decay rate. Here, we use stacks of aftershocks to capture signals over more than two decades in time. Following geophysical studies^5^,^34^,^35^, the definitions of mainshocks and aftershocks as well as the declustering technique are described in Fig. 5 and in the Methods section.

**Figure 6:**
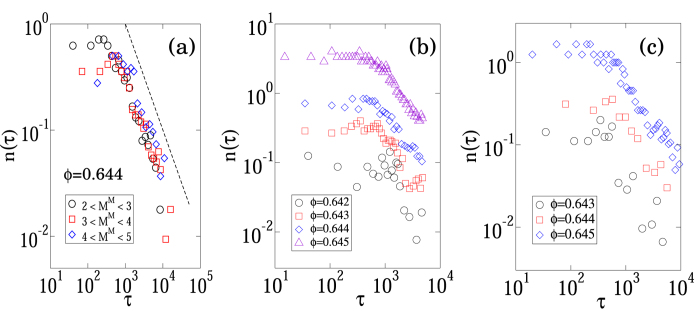
The aftershock decay rate. (**a**) Occurence rate of magnitude *M*^A^ ∈ [1, 3] aftershocks for different mainshock magnitude ranges and a volume fraction *ϕ* = 0.644. The dashed line is proportional to 1/*t* (i.e., the Omori Law). (**b**) Occurence rate of *M*^A^ ∈ [1, 3] aftershocks for different volume fractions and magnitude *M*^M^ ∈ [3, 4] mainshocks. (**c**) Occurence rate of *M*^A^  ∈ [2, 4] aftershocks for different volume fractions and magnitude *M*^M^ ∈ [4, 5]. In all cases, the shear rate 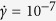 and individual aftershocks are stacked according to their main shock times to compensate for the small number of events in each sequence. There are at least 10 mainshocks and 1000 aftershocks in each sequence. Note the time delay before the onset of a power-law aftershock decay rate.

Figure 6a shows the aftershock rates with respect to the time *τ* from the mainshock for several ranges of mainshock magnitude, which is denoted by *M*^M^. The magnitude of aftershocks is denoted by *M*^A^. The studied ranges of *M*^M^ and *M*^A^-values are chosen so that we have enough events in the numerical output to get sufficient statistics. We find that the MOL (Eq. 2) holds with the exponent *p* ≃ 1 irrespective of the magnitude range of mainshocks. Figure 6b,c show the dependence of the aftershock rate on the volume fraction. We test several volume fractions (*ϕ* = {0.642, 0.643, 0.644, 0.645} and shear rate 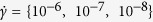) to verify the existence of the MOL irrespective of these parameters. This relaxation process, characterised by an initial plateau followed by a power law decay, remains stable across the entire parameter space of the model. This is not the case for the GR law (Fig. 3). In addition, the time constant *c* is insensitive to the volume fraction, as shown in Fig. 6b,c.

If aftershocks are triggered by external shear, aftershock statistics may be controlled by the shear strain that is applied after a mainshock, 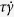. In this case the aftershock decay rate *n*(*τ*) must be scaled as 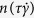. Taking the MOL into account, this means that 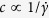. However, we confirm that the *c*-value is independent of the shear rate. This indicates that aftershocks are not driven by the additional shear strain applied after a mainshock; rather, they are caused by the intrinsic relaxation dynamics. Thus, we do not expect aftershocks in the quasi-static shear deformation, in which a system fully relaxes to a stable configuration after an avalanche.

**Figure 7:**
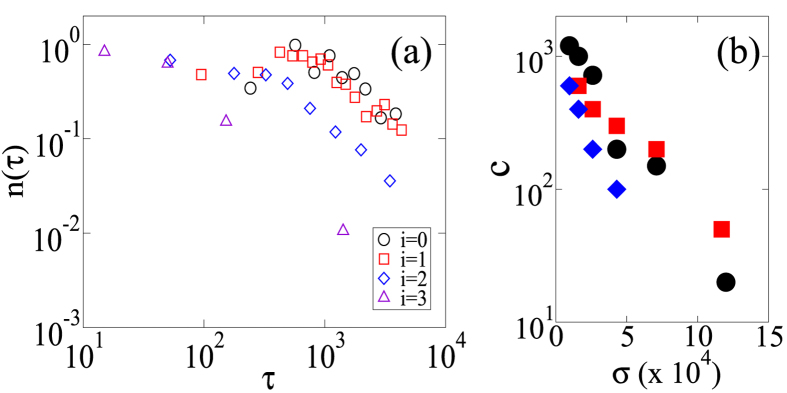
Dependency of the aftershock decay rate on the global shear stress. (**a**) Occurence rate of *M*^A^ ∈ [1, 3] aftershocks for different ranges of shear stress value and a volume fraction *ϕ* = 0.644. Aftershocks *i* ∈ {0, 1, 2, 3} are classify according to the global shear stress value *σ*_*i*_ ∈ [exp(*i* − 10), exp(*i* − 9)] at the inititation of the avalanches. The time delay before the onset of the power-law decay rate is systematically decreasing with the level of stress (i.e., an increasing *i*-value). (**b**) Negative dependence of the *c*-value on the global shear stress. Circles: *ϕ* = 0.644, *M*^M^ ∈ [3, 4], and *M*^A^ ∈ [1, 3]; Squares: *ϕ* = 0.645, *M*^M^ ∈ [3, 4], and *M*^A^ ∈ [1, 3]. Diamonds: *ϕ* = 0.644, *M*^M^ ∈ [2, 3], and *M*^A^ ∈ [1, 2].

Next, we analyse how the time constant *c* depends on the magnitude of the shear stress. We define for aftershocks the stress range [*σ*_min_, *σ*_max_] and select only aftershocks that belong to this stress range and the magnitude range 
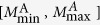
. The aftershock rates are shown in Figure 7a, in which the MOL (with *p* = 1) holds clearly and *c* is a decreasing function of shear stress. We estimate the *c*-values by fitting the data with *A*/(*τ* + *c*) using the maximum-likelihood method. As shown in Fig. 7b, the *c*-value has a negative dependence on the shear stress. We confirm this stress dependence for two volume fractions (*ϕ* = {0.644, 0.645}) and for two magnitude ranges (*M*^M^ ∈ [3, 4], *M*^A^ ∈ [1, 3] and *M*^M^ ∈ [4, 5], *M*^A^ ∈ [2, 4]). This negative shear-stress dependence of the *c*-value for aftershocks is consistent with the trend inferred from seismological observations^5^,^34^,^35^.

## Discussion and conclusion

Many systems exhibit crackling noise behaviours that share common properties with earthquake statistics^36^. However, none of them as yet been able to systematically capture the dependence of both the GR law and the MOL on the level of stress^5^. Here, we have not only shown that these two fundamental laws of statistical seismology are relevant to avalanches in sheared granular matter, but also they have common dependence on the level of stress.

Concerning the GR law, which is commonly observed in a wide range of materials under different conditions from laboratory experiments to the field, we find scale invariance in the model only for a narrow range of volume fraction (*ϕ* ≃ 0.644). This does not contradict the previous studies, most of which involve different boundary conditions. For example, under constant pressure condition^13^,^15^, a system can be self-organised to a critical volume fraction at sufficiently low shear rates. Additionally, in some experiments conducted at constant volume fractions, the GR law hardly approximates avalanche-size distributions if the volume fraction is lower than a critical value^16^,^17^. In contrast, the GR law holds for earthquakes irrespective of the details: tectonic setting, depth, rock type, and loading rate. We do not have a clear answer about this difference. Nevertheless, the fact that our model can produce self-similar scalings over more than five orders of magnitude at the critical volume fraction is sufficient to differentiate between the GR law and other types of distribution. Hence, we can explore the sensibility of this power-law regime to different conditions, especially to shear stress. In addition, when the GR law is not respected, a characteristic avalanche size emerges, a behaviour that may be compared to the characteristic earthquake distribution^37^,^38^.

Concerning aftershocks, we have shown that similar relaxation process may occur in sheared granular media and in the seismogenic crust. In addition, the negative stress dependences of the time delay before the onset of the power-law decay rate is the same as the one observed in real seismicity. This stress dependence is also coincident with the behaviour of some simple models based on a frictional constitutive law^39^, damage mechanics^34^, or static fatigue^40^,^41^, despite the absence of such physical processes in our granular model. A quantitative result in the present study, that the time constant *c* in MOL is an exponentially decreasing function of the applied shear stress, should be tested in more realistic and complex systems that are relevant to seismogenic zones as well as various industrial situations.

## Methods

### Simulation model

Our three-dimensional granular system is made of frictionless spheres with diameters of *d* and 0.7*d* (the ratio of populations is 1:1). For the sake of simplicity, we assume that the mass *m* of these particles are the same. We limit ourselves to a rather small-size system (*N* = 1500) for computational efficiency. Using the radius and the position of particle *i*, which are denoted by *R*_*i*_ and *r*_*i*_, respectively, the force between particles *i* and *j* is written as 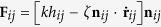. Here **r**_*ij*_ = **r**_*i*_ − **r**_*j*_, **n**_*ij*_ = **r**_*ij*_/|**r**_*ij*_|, and *h*_*ij*_ = (*R*_*i*_ + *R*_*j*_) − |**r**_*ij*_| is the overlap length. If *R*_*i*_ + *R*_*j*_ < |**r**_*ij*_|, particles *i* and *j* are not in contact so that the force vanishes.

Throughout this study, we adopt the units in which *d* = 1, *m* = 1, and *k* = 1. We choose *ζ* = 2.0, which corresponds to the vanishing coefficient of restitution. A constant shear rate 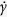 is applied to the system through the Lees-Edwards boundary conditions^42^. Note that under these boundary conditions the system volume is constant. Thus, the important parameters are the shear rate 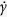 and the packing fraction *ϕ*.

To set-up the initial condition, particles are randomly distributed in a simulation box with zero shear stress. When the kinetic energy has relaxed to zero, a constant shear rate is applied. To avoid transient behaviours, we concentrate only on the data for which the the total strain is greater than 100%. Simultaneously, we verify that a steady state of uniform shear rate and uniform volume fraction is achieved. Here, we investigate such uniform steady states only.

### Definition of avalanche

The beginning of an avalanche is taken at time *t* = *t*_1_ at which the total elastic energy *E*(*t*) starts decreasing; i.e., 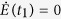 and 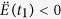. Symmetrically, the end of an event is the time *t* = *t*_2_ at which 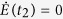 and 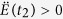. We also calculate the so-called global shear stress 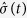 using the virial^43^. In analysing the time series, we did not set the noise threshold. Namely, any small increase in energy leads to the termination of an avalanche.

For simplicity, the spatial information of avalanches is discarded. In this case, one might overlook simultaneous avalanches occurring at different places. However, this is unlikely because the present system is small (i.e. the characteristic length is approximately of 9*d*).

### Definitions of mainshocks and aftershocks

Because aftershocks result from changes of stress induced by a mainshock, we disregard the smaller avalanches for which *M* < 0 and consider ranges of magnitude for mainshocks 
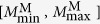
 and aftershocks 
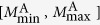
. In practice, a magnitude 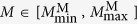 event occurring at time *t*_M_ is not a mainshock if there is at least one avalanche of the same or higher magnitude range in the time interval [*t*_M_ − Δ*T*, *t*_M_ + Δ*T*]. Thus, we only consider isolated mainshocks and, taking sufficiently large Δ*T*-values, we also avoid overlapping aftershock sequences that belong to different mainshocks. Then, all 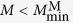 avalanches that follow a magnitude *M*^M^ mainshock within the time interval [*t*_*M*_, *t*_*M*_ + Δ*T*] are regarded as aftershocks. Finally, each aftershock is characterised by its magnitude *M*^A^ and the elapsed time *τ* since the mainshock.

In order to reduce artefacts related to event detectability, we disregard larger magnitude range for mainshocks and smaller magnitude range for aftershocks. In addition, the magnitude range of mainshocks are chosen within the intermediate magnitude range in which the GR law holds. Using this strict methodology, we considerably reduce the number of events in our artificial catalogues. Therefore, in all ranges of magnitudes, aftershocks are stacked with respect to the time of their mainshocks to finally end-up with a single aftershock sequence.

## Additional Information

**How to cite this article**: Hatano, T. *et al*. Common dependence on stress for the statistics of granular avalanches and earthquakes. *Sci. Rep*. **5**, 12280; doi: 10.1038/srep12280 (2015).

## Figures and Tables

**Figure 1:**
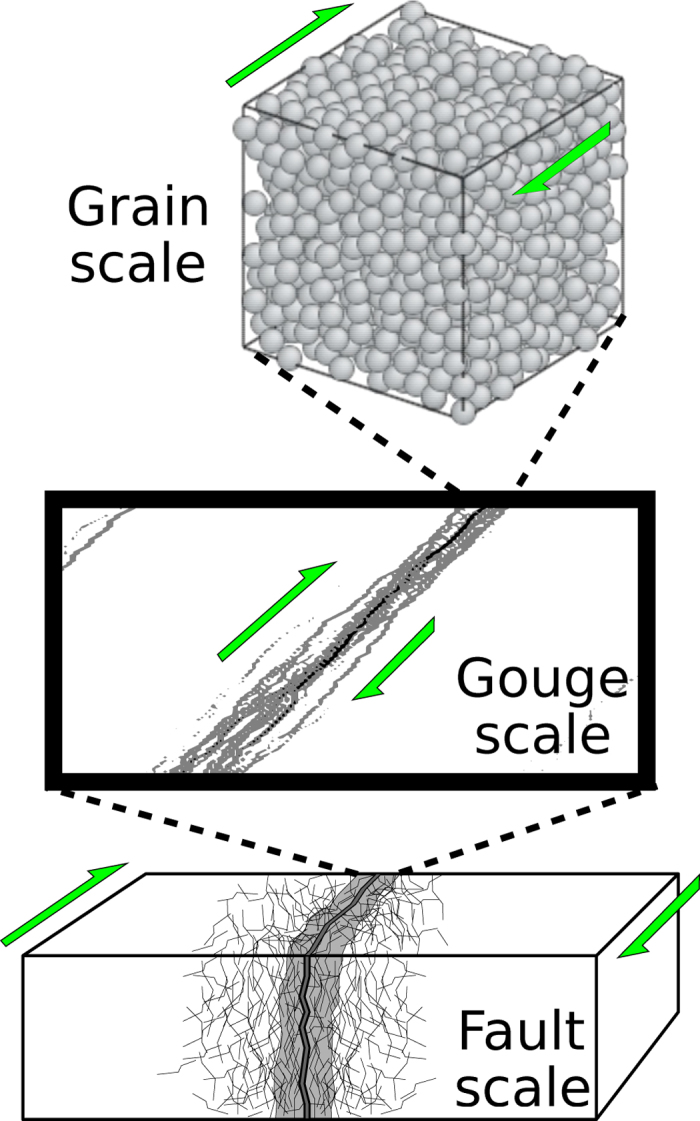
A conceptual model of a fault zone using a 3D granular system. We consider that the fault zone is a strongly damaged area that may be investigated through granular mechanics. The granular model represents a thin gouge layer. The constant grain size, the relatively high porosity and the low grain size to system size ratio of the model do not capture the natural structural and compositional complexities of fault zones in nature. Despite these strong differences, we explore similarities between the dynamics of avalanches in the model and earthquakes.

## References

[b1] Gutenberg B. & Richter C. Frequency of earthquakes in California. Bull. Seism. Soc. Am. 34, 185–188 (1944).

[b2] Omori F. On after-shocks of earthquakes. J. Coll. Sci. Imp. Univ. Tokyo 7, 111–200 (1894).

[b3] Utsu T. Aftershocks and earthquake statistics (1): some parameters which characterize an aftershock sequence and their interrelations. J. Facul. Sci. Hokkaido Univ. Ser. VII 3, 129–195 (1970).

[b4] Schorlemmer D., Wiemer S. & Wyss M. Variations in earthquake-size distribution across different stress regimes. Nature 437, 539–542 (2005). 16177788 10.1038/nature04094

[b5] Narteau C., Byrdina S., Shebalin P. & Schorlemmer D. Common dependence on stress for the two fundamental laws of statistical seismology. Nature 462, 642–645 (2009). 19956258 10.1038/nature08553

[b6] Mogi K. Magnitude-frequency relation for elastic shocks accompanying fractures of various materials and some related problems in earthquakes (2nd Paper). Bull. Earthquake Res. Inst. 40, 831–853 (1962).

[b7] Scholz C. H. The frequency-magnitude relation of microfracturing in rock and its relation to earthquakes. Bull. Seism. Soc. Am. 58, 399–415 (1968).

[b8] Hirata T. Omori’s Power Law aftershock sequences of microfracturing in rock fracture experiment. J. Geophys. Res. 92, 6215–6221 (1987).

[b9] Bar J. . Statistical similarity between the compression of a porous material and earthquakes. Phys. Rev. Lett. 110, 088702 (2013). 23473208 10.1103/PhysRevLett.110.088702

[b10] Turcotte D. L. Self-organized criticality. Rep. Prog. Phys. 62, 1377–1429 (1999).

[b11] Narteau C. Classification of seismic patterns in a hierarchical model of rupture a new phase diagram for seismicity. Geophys. Journ. Int. 168, 710–722 (2007).

[b12] Dalton F. & Corcoran D. Self-organized criticality in a sheared granular stick-slip system. Phys. Rev. E 63, 061312 (2001). 10.1103/PhysRevE.63.061312 11415097

[b13] Dalton F. & Corcoran D. Basin of attraction of a bounded self-organized critical state. Phys. Rev. E 65, 031310 (2002). 10.1103/PhysRevE.65.031310 11909049

[b14] Peña A., McNamara S., Lind P. G. & Herrmann H. J. Avalanches in anisotropic sheared granular media. Granul. Matter 11, 243–252 (2009).

[b15] Bretz M., Zaretzki R., Field S. B., Mitarai N. & Nori F. Broad distribution of stick-slip events in Slowly Sheared Granular Media: Table-top production of a Gutenberg-Richter-like distribution. EPL 74, 1116–1122 (2006).

[b16] Twardos M. & Dennin M. Slow steady-shear of plastic bead rafts. Granul. Matter 7, 91–96 (2005).

[b17] Behringer R. P., Bi D., Chakraborty B. Henkes S. & Hartley R. R. Why Do Granular Materials Stiffen with Shear Rate? Test of Novel Stress-Based Statistics. Phys. Rev. Lett. 101, 268301 (2008). 19437678 10.1103/PhysRevLett.101.268301

[b18] Kun F., Varga I., Lennartz-Sassinek S. & Main I. G. Approach to failure in porous granular materials under compression. Phys. Rev. E 88, 062207 (2013). 10.1103/PhysRevE.88.062207 24483436

[b19] Kun F., Varga I., Lennartz-Sassinek S. & Main I. G. Rupture cascades in a discrete element model of a porous sedimentary rock. Phys. Rev. Lett. 112, 065501 (2014). 24580692 10.1103/PhysRevLett.112.065501

[b20] Mora P. & Place D. Simulation of the frictional stick-slip instability. Pure Appl. Geophys. 143, 61–87 (1994).

[b21] Aharonov E. & Sparks D. Stick-slip motion in simulated granular layers. J. Geophys. Res. 109, B09306 (2004).

[b22] Johnson P. A. & Jia X. Nonlinear dynamics, granular media and dynamic earthquake triggering. Nature 437, 871–874 (2005). 16208368 10.1038/nature04015

[b23] Durian D. J. Bubble-scale model of foam mechanics: Melting, nonlinear behavior, and avalanches. Phys. Rev. E 55, 1739–1751 (1997).

[b24] Tewari S. . Statistics of shear-induced rearrangements in a two-dimensional model foam. Phys. Rev. E 60, 4385–4396 (1999). 10.1103/physreve.60.4385 11970293

[b25] Kabla A., Scheibert J. & Debregeas G. Quasi-static rheology of foams. Part 2. Continuous shear flow. J. Fluid Mech. 587, 45–72 (2007).

[b26] Maloney C. & Lematre A. Subextensive scaling in the athermal, quasistatic limit of amorphous matter in plastic shear flow. Phys. Rev. Lett. 93, 16001 (2004).

[b27] Lerner E. & Procaccia I. Density scaling of avalanche statistics in amorphous solids. arXiv:1004.3193v1 (2010).

[b28] Heussinger C., Chaudhuri P. & Barrat J.-L. Fluctuations and correlations during the shear flow of elastic particles near the jamming transition. Soft Matter 6, 3050–3058 (2010).

[b29] Bailey N. P., Schiotz J., Lematre A. & Jacobsen K. W. Avalanche size scaling in sheared three-dimensional amorphous solid. Phys. Rev. Lett. 98, 095501 (2007). 17359166 10.1103/PhysRevLett.98.095501

[b30] Salerno K. M., Maloney C. E. & Robbins M. O. Avalanches in strained amorphous solids : Does inertia destroy critical behavior? Phys. Rev. Lett. 109, 105703 (2012). 23005301 10.1103/PhysRevLett.109.105703

[b31] Hanks T. C. & Kanamori H. A moment magnitude scale. J. Geophys. Res. 84, 2348–2350 (1979).

[b32] Ferenc K., Lenkey G., Takács N. & Beke D. Structure of magnetic noise in dynamic fracture. Phys. Rev. Lett. 93, 227204 (2004). 15601114 10.1103/PhysRevLett.93.227204

[b33] Ciliberto S. & Laroche C. Experimental evidence of self organized criticality in the stick-slip dynamics of two rough elastic surfaces. J. Phys. I France 4, 223–235 (1994).

[b34] Narteau C., Shebalin P. & Holschneider M. Temporal limits of the power law aftershock decay rate. J. Geophys. Res. 107, B122359 (2002).

[b35] Narteau C., Shebalin P. & Holschneider M. Onset of power law aftershock decay rates in southern California. Geophys. Res. Lett. 32, L22312 (2005).

[b36] Sethna J. P., Dahmen K. A. & Myers C. R. Crackling noise. Nature 410, 242–250 (2001). 11258379 10.1038/35065675

[b37] Schwartz D. & Coppersmith K. Fault behavior and caracteristic earthquake: examples from the Wasatch and San Andreas fault zones. J. Geophys. Res. 109, 5681–5698 (1984).

[b38] Naylor M., Greenhough J., McCloskey J., Bell a. F. & Main I. G. Statistical evaluation of characteristic earthquakes in the frequency-magnitude distributions of Sumatra and other subduction zone regions. Geophys. Res. Lett. 36, L20303 (2009).

[b39] Dieterich J. A constitutive law for rate of earthquake production and its application to earthquake clustering. J. Geophys. Res. 99, 2601–2618 (1994).

[b40] Shcherbakov R. & Turcotte D. L. A damage mechanics model for aftershocks. Pure Appl. Geophys. 161, 2379–2391 (2004).

[b41] Ben-Zion Y. & Lyakhovsky V. Analysis of aftershocks in a lithospheric model with seismogenic zone governed by damage rheology. Geophys. J. Int. 165, 197–210 (2006).

[b42] Allen M. P. & Tildesley D. J. Computer simulation of liquids (Oxford University Press, New York, 1987).

[b43] Zubarev D. N. Nonequilibrium statistical thermodynamics (Consultants Bureau, New York, 1974).

